# Regorafenib for Taiwanese patients with unresectable hepatocellular carcinoma after sorafenib failure: Impact of alpha‐fetoprotein levels

**DOI:** 10.1002/cam4.4430

**Published:** 2021-11-16

**Authors:** Po‐Yao Hsu, Tzu‐Sheng Cheng, Shih‐Chang Chuang, Wen‐Tsan Chang, Po‐Cheng Liang, Cheng‐Ting Hsu, Yu‐Ju Wei, Tyng‐Yuan Jang, Ming‐Lun Yeh, Ching‐I Huang, Yi‐Hung Lin, Chih‐Wen Wang, Ming‐Yen Hsieh, Nai‐Jen Hou, Meng‐Hsuan Hsieh, Yi‐Shan Tsai, Yu‐Min Ko, Ching‐Chih Lin, Kuan‐Yu Chen, Chia‐Yen Dai, Zu‐Yau Lin, Shinn‐Cherng Chen, Jee‐Fu Huang, Wan‐Long Chuang, Chung‐Feng Huang, Ming‐Lung Yu

**Affiliations:** ^1^ Graduate Institute of Clinical Medicine College of Medicine Kaohsiung Medical University Kaohsiung Taiwan; ^2^ Hepatobiliary Division Department of Internal Medicine Kaohsiung Medical University Hospital Kaohsiung Medical University Kaohsiung Taiwan; ^3^ Department of Surgery Kaohsiung Medical University Hospital Kaohsiung Medical University Kaohsiung Taiwan; ^4^ Department of Internal Medicine Kaohsiung Municipal Ta‐Tung Hospital Kaohsiung Medical University Kaohsiung Taiwan; ^5^ School of Medicine and Hepatitis Research Center College of Medicine, and Center for Liquid Biopsy and Cohort Research and Center for Cancer Research Kaohsiung Medical University Kaohsiung Taiwan; ^6^ Institute of Biomedical Sciences National Sun Yat‐Sen University Kaohsiung Taiwan; ^7^ National Pingtung University of Science and Technology Pingtung Taiwan

**Keywords:** efficacy, hepatocellular carcinoma, regorafenib, sorafenib

## Abstract

**Background and Aims:**

Regorafenib has demonstrated its survival benefit for unresectable hepatocellular carcinoma (uHCC) patients in a phase III clinical trial. We aimed to assess the efficacy and tolerability of regorafenib and the predictors of treatment outcomes in Taiwanese patients.

**Methods:**

We analyzed the survival, best overall response, predictors of treatment outcomes, and safety for uHCC patients who had tumor progression on sorafenib therapy and received regorafenib as salvage therapy between March 2018 and November 2020.

**Results:**

Eighty‐six patients with uHCC were enrolled (median age, 66.5 years; 76.7% male). The median regorafenib treatment duration was 4.0 months (95% confidence interval [CI], 3.6–4.6). The most frequently reported adverse events were hand‐foot skin reaction (44.2%), diarrhea (36.0%), and fatigue (29.1%). No unpredictable toxicity was observed during treatment. The median overall survival (OS) with regorafenib was 12.4 months (95% CI, 7.8–17.0) and the median progression‐free survival (PFS) was 4.2 months (95% CI, 3.7–4.7). Of 82 patients with regorafenib responses assessable, 4 patients (4.9%) achieved a partial response, and 33 (40.2%) had stable disease, leading to a disease control rate (DCR) of 45.1% (*n* = 37). Patients possessing baseline AFP < 400 ng/ml exhibited a markedly longer median OS, median PFS, and higher DCR compared with their counterparts (15.7 vs. 8.1 months, 4.6 vs. 3.7 months, 60.9% vs. 27.5%, respectively). Despite possessing high baseline AFP levels, patients with early AFP response (>10% reduction at 4 weeks or >20% reduction at 8 weeks after regorafenib administration) exhibited comparable treatment outcomes to those with baseline AFP < 400 ng/ml.

**Conclusions:**

The results of this real‐world study verified the tolerability and efficacy of regorafenib treatment for uHCC patients who failed prior sorafenib therapy, especially for those with lower baseline AFP levels or with early AFP response.

## Graphical abstract

This real‐world study verified the safety and efficacy of regorafenib for Taiwanese patients with unresectable HCC. Patients with lower baseline AFP levels or with early AFP response exhibited a markedly better survival outcome and higher disease control rate.

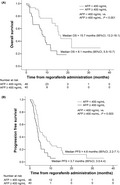

## 1 INTRODUCTION

Hepatocellular carcinoma (HCC) is one of the main causes of cancer‐related death globally. The World Health Organization estimates that more than 1 million patients will die from HCC by 2030.^1^ HCC generally arises from underlying liver diseases, such as hepatitis B virus (HBV) and hepatitis C virus (HCV) infection and alcohol abuse, leading to substantial clinical and economic burdens. Therapeutic strategies for HCC are largely determined by the tumor stage and remaining liver function. For patients with advanced disease or who have intermediate‐stage disease with refractory/poor response to TACE, systemic therapy is the main treatment method.^2^, ^3^, ^4^

Sorafenib, a multikinase inhibitor, was approved as the first‐line systemic therapy for advanced HCC based on the phase III SHARP trial in 2007.^5^ Recently, substantial progress has been made in developing new effective systemic therapies for HCC. Regorafenib is also a multikinase inhibitor that targets vascular endothelial growth factor receptors and several kinases to protect against angiogenesis, oncogenesis, and tumor metastasis.^6^, ^7^ In the randomized placebo‐controlled phase III RESORCE trial, regorafenib increased survival, as compared with placebo, from 7.8 to 10.6 months in patients who experienced tumor progression on sorafenib treatment.^8^ Thus, regorafenib has become the first second‐line agent approved by the United States Food and Drug Administration and is a recommended therapy for advanced HCC following sorafenib failure.^2^, ^3^, ^9^

Although several second‐line regimens, including cabozantinib, ramucirumab, nivolumab plus ipilimumab, and pembrolizumab, are now available for the management of advanced HCC previously treated with sorafenib, the status of approval and reimbursement of each agent differ among countries.^10^, ^11^, ^12^, ^13^ In Taiwan, regorafenib is the only secondary line therapy covered by the National Health Insurance for advanced HCC progressing on sorafenib before May 2021. However, real‐world data regarding the effectiveness and safety of regorafenib for Taiwanese patients remain limited. The current study aimed to investigate the efficacy and safety, and to explore the potential early predictors of regorafenib therapy for unresectable HCC (uHCC) after sorafenib failure in real‐world clinical settings.

## 2 MATERIALS AND METHODS

### 2.1 Study population

Consecutively recruited HCC patients who received regorafenib were enrolled between March 2018 and November 2020 at Kaohsiung Medical University Hospital. The diagnosis of HCC was made according to the criteria of the American Association for the Study of Liver Diseases. The diagnosis was also confirmed by an HCC expert group for each patient. All patients with HCC were eligible for inclusion in this study once they met the following criteria: (1) HCC progressed during prior first‐line sorafenib therapy; (2) at least one dose of regorafenib was given after confirmed tumor progression on sorafenib treatment; (3) Barcelona Clinic Liver Cancer (BCLC) stage B or C disease, and (4) Child–Pugh class A at the time of regorafenib initiation.

### 2.2 Regorafenib treatment

The standard starting dose of regorafenib was 160 mg orally once a day for 3 weeks, followed by 1 week of no treatment in each cycle. Nevertheless, modification of the starting dose was allowed at the discretion of the attending physicians. Regorafenib therapy was continued until disease progression, death, or any intolerable adverse event. Doses were decreased or interrupted according to the protocol of the RESORCE trial.^8^

### 2.3 Assessment

All data were collected retrospectively and analyzed. We analyzed the clinical parameters including baseline characteristics of patients (age, sex, etiology of liver cancer, albumin‐bilirubin [ALBI] score, BCLC stage, alpha‐fetoprotein [AFP], radiological assessment, and data in regard to sorafenib therapy), treatment prior to and post regorafenib, and adverse events during regorafenib administration. OS was defined as the time from the initiation of sorafenib and regorafenib, respectively, to death from any cause. PFS was defined as the time from the initiation of regorafenib to disease progression or death from any cause. The census of survival status was checked by the end of February 2021. Time to progression (TTP) was defined as the time from the initiation of treatment with sorafenib/regorafenib to the date of disease progression. Response was evaluated and classified as complete response (CR), partial response (PR), stable disease (SD), and progressive disease (PD) according to the Response Evaluation Criteria in Solid Tumors version 1.1 (RECIST v1.1) using dynamic computed tomography or dynamic magnetic resonance imaging every 6–12 weeks. Disease control rate (DCR) was defined as either tumor response (CR + PR) or SD. The best overall response was the best response recorded from the start of sorafenib/regorafenib until disease progression. Tolerability was assessed during every visit to the clinic and was graded based on the National Cancer Institute Common Terminology Criteria for Adverse Events version 5.0. To address the survival benefit of regorafenib, another consecutive group consisting of age‐, sex‐, Child–Pugh status‐, BCLC stage‐, and AFP level (≥400 or <400 ng/ml)‐matched patients who received sorafenib alone^14^ was selected as a historical control with a 1:1 propensity score matching ratio. We compared the OS after sorafenib discontinuation between patients in the current study and those in the historical control group.

### 2.4 Statistical analysis

The clinical characteristics of patients were presented using the median (range), median [inter‐quartiles], and number (percentage). The result of safety analysis was described as the number (percentage). The Kaplan–Meier method, the log‐rank test, and the Cox proportional hazard model were used for survival analyses. Variables with a potential relationship (*p* < 0.1) identified in the univariate analyses were included in the multivariate analysis. The chi‐squared test or the Fisher's exact test was used to compare the DCRs of different groups. Logistic regression was used to identify the independent prognostic factors for DCR by univariate or multivariate model. We performed a 1:1 match for each patient using propensity score matching to select historical controls. Eighty‐one pairs of patients were successfully matched using the following five covariates: age, sex, Child–Pugh status, BCLC stage, and AFP level (≥400 vs. <400 ng/ml). All *p* values are two‐sided, and *p* < 0.05 is determined as statistically significant difference. All database processing and analyses were conducted with the SPSS version 24.0 (SPSS, Inc., Chicago, IL, USA).

## 3 RESULTS

### 3.1 Patient characteristics

#### 3.1.1 Baseline characteristics at initiation of regorafenib

A total of 86 patients who underwent regorafenib treatment for uHCC were enrolled in this study. The baseline characteristics are summarized in Table 1. HBV infection was the most common etiology of HCC (*n* = 41, 47.7%), followed by HCV infection (*n* = 24, 27.9%), non‐B, non‐C status (*n* = 15, 17.4%), and HBV/HCV dual infection (*n* = 6, 7.0%). Sixty‐six patients (76.7%) were male. At the initiation of regorafenib administration, the median age was 66.5 years (range, 39–89). Most patients had BCLC stage C (*n* = 75, 87.2%). Macrovascular invasion occurred in 34 patients (39.5%) and 38 patients (44.2%) whose largest tumor was ≥5 cm. Fifty‐one patients (59.3%) had extrahepatic metastasis. The most common metastatic sites were lung (*n* = 22, 25.6%), bone (*n* = 17, 19.8%), and adrenal glands (*n* = 3, 3.5%). Forty patients (46.5%) had baseline AFP levels ≥400 ng/ml.

**TABLE 1:** Characteristics of the HCC patients

Characteristics	At initiation of sorafenib *N *= 86	At initiation of regorafenib *N* = 86
Age (years), median (range)	66.0 (38–88)	66.5 (39–89)
Male, *n* (%)	66 (76.7)	
Etiology, *n* (%)		
Hepatitis B	41 (47.7)	
Hepatitis C	24 (27.9)	
Both hepatitis B and C	6 (7.0)	
Non‐hepatitis B and C	15 (17.4)	
Child–Pugh score, *n* (%)		
A	86 (100)	86 (100)
ALBI score, *n* (%)		
Grade 1	65 (75.6)	54 (62.8)
Grade 2	21 (24.4)	32 (37.2)
BCLC stage, *n* (%)		
B	12 (14.0)	11 (12.8)
C	74 (86.0)	75 (87.2)
Largest tumor size ≥5 cm, *n* (%)	31 (36.0)	38 (44.2)
Macrovascular invasion, *n* (%)	34 (39.5)	34 (39.5)
Extrahepatic metastasis, *n* (%)	39 (45.3)	51 (59.3)
Lung	14 (16.3)	22 (25.6)
Bone	11 (12.8)	17 (19.8)
Adrenal gland	1 (1.2)	3 (3.5)
Others	15 (17.4)	20 (23.3)
AFP ≥400 ng/ml, *n* (%)	38 (44.2)	40 (46.5)
Prior treatment before sorafenib, *n* (%)	64 (74.4)	
TACE	42 (48.8)	
Surgery	32 (37.2)	
Local ablation	15 (17.4)	
Radiation therapy	3 (3.5)	
Concurrent treatment, *n* (%)	55 (64.0)	22 (25.6)
TACE	30 (34.9)	10 (11.6)
Radiation therapy	28 (32.6)	9 (10.5)
Local ablation	6 (7.0)	0 (0)
Pembrolizumab	2 (2.3)	2 (2.3)
Nivolumab	0 (0)	2 (2.3)
Other	5 (5.8)	0 (0)
Treatment after regorafenib, (%)		36 (41.9)
Lenvatinib		14 (16.3)
TACE		9 (10.5)
Radiation therapy		8 (9.3)
Atezolizumab + Bevacizumab,		4 (4.7)
Nivolumab		2 (2.3)
Cabozantinib		1 (1.2)
Others		14 (16.3)
Median duration of therapy, months [inter‐quartiles]	5.5 [3.5; 12.8]	4.0 [2.7; 7.2]
Median time from sorafenib discontinuation to regorafenib administration, months [inter‐quartiles]	0.03 [0.03; 1.73]	

Abbreviations: 95% CI, 95% confidence interval; AFP, alpha‐fetoprotein; ALBI score, albumin‐bilirubin score; BCLC stage, Barcelona Clinic Liver Cancer stage; HCC, hepatocellular carcinoma; TACE, transarterial chemoembolization.

#### 3.1.2 Baseline characteristics at initiation of sorafenib

The patient characteristics at initiation and clinical data during regorafenib treatment are listed in Tables 1 and 2. Only 22 HCC patients (25.6%) took sorafenib as the first‐line therapy after the diagnosis of HCC, and the most frequent treatments before sorafenib were TACE, surgery, local ablation, and radiation therapy. All patients received sorafenib as the first‐line systemic therapy before the administration of regorafenib, and all patients discontinued sorafenib due to tumor progression. No patient achieved CR during sorafenib treatment. The best response to sorafenib was PR in seven patients (8.1%). Forty‐nine patients (57.0%) had SD, leading to a DCR of 65.1% (*n* = 56) with the use of sorafenib. The median TTP on sorafenib was 5.5 months (95% CI, 3.2–7.8) (Table 2) and the median sorafenib treatment duration was 5.5 months [3.5; 12.8]. In addition, the median time between sorafenib discontinuation and regorafenib initiation was 0.03 months [0.03; 1.73].

**TABLE 2:** Treatment efficacy of first‐line sorafenib and second‐line regorafenib therapy

Variable	Total (*n* = 86)
Prior sorafenib	
Best overall response by RECIST version 1.1, *n* (%)	
Complete response	0 (0)
Partial response	7 (8.1)
Stable disease	49 (57.0)
Progressive disease	30 (34.9)
Objective response rate, *n* (%)	7 (8.1)
Disease control rate, *n* (%)	56 (65.1)
Time to progression, median months (95% CI)	5.5 (3.2–7.8)
Regorafenib^a^	
Best overall response by RECIST version 1.1, *n* (%)	
Complete response	0 (0)
Partial response	4 (4.9)
Stable disease	33 (40.2)
Progressive disease	45 (54.9)
Objective response rate, *n* (%)	4 (4.9)
Disease control rate, *n* (%)	37 (45.1)
Time to progression, median months (95% CI)	4.2 (3.7–4.8)
Survival	
Overall survival on regorafenib, median months (95% CI)	12.4 (7.8–17.0)
Progression‐free survival on regorafenib, median months (95% CI)	4.2 (3.7–4.7)
Overall survival since sorafenib administration, median months (95% CI)	28.2 (18.5–37.8)

Abbreviations: 95% CI, 95% confidence interval; RECIST, Response Evaluation Criteria in Solid Tumors.

^a^ Available imaging tests for response assessment in 82 patients.

### 3.2 Efficacy of regorafenib

**FIGURE 1:**
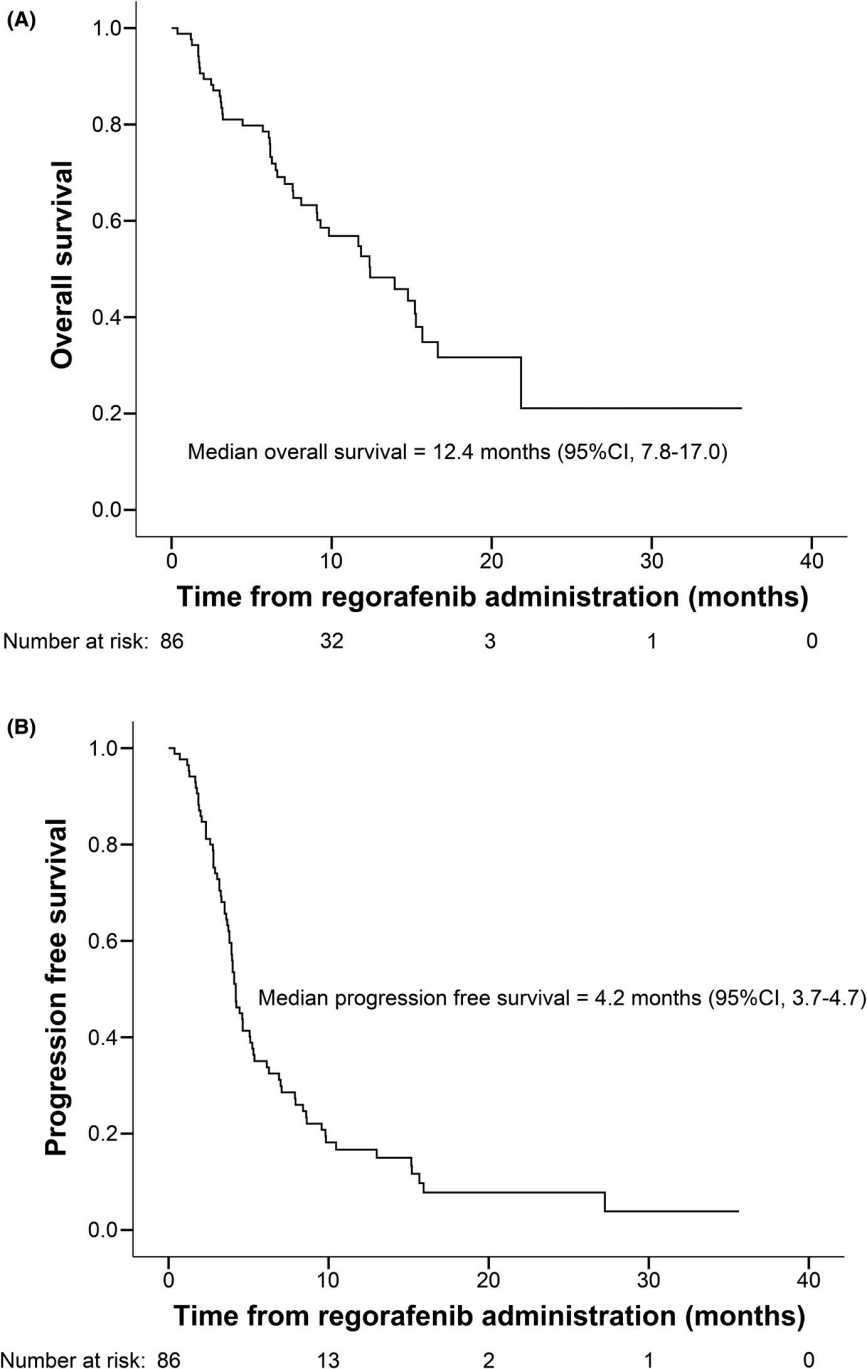
The Kaplan–Meier analysis of overall survival and progression‐free survival in patients treated with regorafenib. (A) Overall survival with regorafenib and (B) progression‐free survival with regorafenib

The median duration of regorafenib treatment was 4.0 months (95% CI, 3.6–4.6). During a mean follow‐up duration of 9.3 months (range, 0.37–35.6 months), the median OS was 12.4 months (95% CI, 7.8–17.0), the median PFS was 4.2 months (95% CI, 3.7–4.7), and the median TTP was 4.2 months (95% CI, 3.7–4.8) (Figure 1A,B).

Imaging assessments for tumor response were available for 82 patients (95.3%), and the results are presented in Table 2. Four patients (4.9%) achieved PR, and no patients achieved CR. Thirty‐three patients (40.2%) had SD, leading to a DCR of 45.1% (*n* = 37). After discontinuing regorafenib, 36 (41.9%) patients received subsequent anticancer therapy, including lenvatinib in 14 (16.3%) patients, TACE in 9 (10.5%) patients, atezolizumab plus bevacizumab in 4 (4.7%) patients, nivolumab in 2 (2.3%) patients, cabozantinib in 1 (1.2%) patient, and other treatments in 14 (16.3%) patients. Taken together, the median OS since the initiation of sorafenib administration was 28.2 months (95% CI, 18.5–37.8) (Figure S1).

### 3.3 Comparison of OS with historical control of HCC patients treated with sorafenib only

The median OS following sorafenib discontinuation was 15.2 months (95% CI, 13.3–17.2) in patients treated with regorafenib in the present study, which was remarkably longer than that in our previous cohort, which contained patients who received sorafenib alone (3.8 months; 95% CI, 2.8–4.7; *p* < 0.001) (Figure S2A).^14^ After propensity score matching, patients from the present study (sorafenib–regorafenib, *n* = 81) still showed a better median OS after sorafenib discontinuation (15.3 months; 95% CI, 12.5–18.1) than those in the historical control group (*n* = 81; 5.2 months; 95% CI, 2.5–7.9; *p* < 0.001) (Table S1, Figure S2B).

### 3.4 Factor predictive of treatment outcomes on regorafenib

#### 3.4.1 Pretreatment factors

**FIGURE 2:**
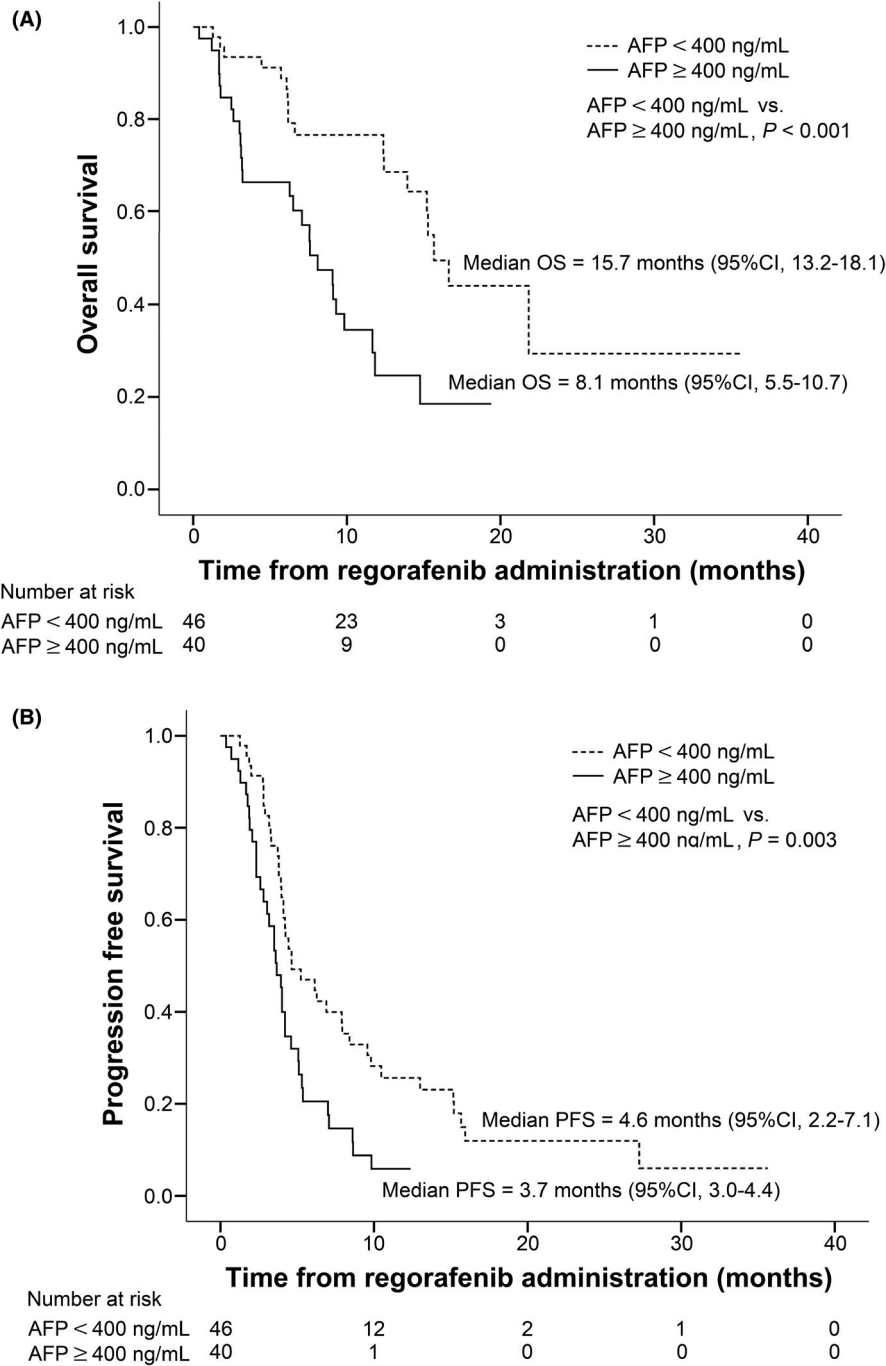
Survival outcomes of regorafenib based on baseline AFP levels (*n* = 86). (A) Overall survival with regorafenib (AFP < 400 vs. ≥400 ng/ml) and (B) progression‐free survival with regorafenib (AFP < 400 vs. ≥400 ng/ml)

We evaluated the prognostic factors associated with survival and disease control rate using the variables of age ≥ 65 years, sex, etiology of HCC, ALBI score, vascular invasion, BCLC stage, largest tumor size ≥5 cm, macrovascular invasion, extrahepatic metastasis, AFP ≥ 400 ng/ml, concurrent treatment with regorafenib, and TTP with sorafenib > median (5.5 months) (Table S2–S5). The independent factors predictive of survival and disease control based on multivariate analysis are summarized in Table 3. HCV infection (vs. NBNC; hazard ratio [HR], 0.35; *p* = 0.037), ALBI grade 2 (vs. grade 1; HR, 3.75; *p* < 0.001), macrovascular invasion (HR, 2.03; *p* = 0.032), and baseline AFP level ≥400 ng/ml (HR, 2.82; *p* = 0.005) were independent factors predictive of OS. Extrahepatic metastasis (HR, 1.99; *p* = 0.007) and baseline AFP level ≥400 ng/ml (HR, 1.82; *p* = 0.035) were independent factors predictive of PFS. Extrahepatic metastasis was the only independent factor predictive of TTP (HR, 2.76; *p* < 0.001). For the best overall response, HCV infection (vs. NBNC; odds ratio [OR], 5.72; *p* = 0.036) and baseline AFP level ≥400 ng/ml (OR, 0.16; *p* = 0.002) were independently associated with the DCR. Considering baseline AFP level was predictive with OS, PFS, and DCR with regorafenib, we further analyzed the treatment outcomes stratified by baseline AFP levels (<400 vs. ≥400 ng/ml). The patients having baseline AFP <400 ng/ml exhibited a markedly longer median OS, median PFS, and higher DCR (15.7, 4.6 months, and 60.9%, respectively) compared with their counterparts (8.1 months, *p* < 0.001; 3.7 months, *p* = 0.003; 27.5%, *p* = 0.002; Figure 2A,B).

**TABLE 3:** Factors predictive of treatment outcomes on regorafenib

	Univariate HR (95% CI)	*p* value	Multivariate HR (95% CI)	*p* value
Overall survival				
HCV (vs. NBNC)	0.55 (0.22–1.34)	0.187	0.35 (0.13–0.94)	0.037
ALBI grade 2 (vs. grade 1)	3.12 (1.70–5.73)	<0.001	3.75 (1.95–7.20)	<0.001
Macrovascular invasion	2.05 (1.12–3.72)	0.019	2.03 (1.06–3.86)	0.032
AFP ≥ 400 ng/ml	2.95 (1.57–5.52)	0.001	2.82 (1.36–5.84)	0.005
Progression‐free survival				
Extrahepatic metastasis	1.84 (1.15–2.94)	0.011	1.99 (1.21–3.28)	0.007
AFP ≥ 400 ng/ml	2.03 (1.25–3.28)	0.004	1.82 (1.04–3.16)	0.035
Time to progression				
Extrahepatic metastasis	2.42 (1.45–4.03)	0.001	2.76 (1.59–4.78)	<0.001

Variables	Univariate OR (95% CI)	*p* value	Multivariate OR (95%CI)	*p* value
Disease control rate^a^				
HCV (vs. NBNC)	3.85 (0.95–15.66)	0.060	5.72 (1.12–29.11)	0.036
AFP ≥ 400 ng/ml	0.25 (0.10–0.63)	0.004	0.16 (0.05–0.52)	0.002

Abbreviations: 95% CI, 95% confidence interval; AFP, alpha‐fetoprotein; ALBI score, albumin‐bilirubin score; HCV, hepatitis C virus; HR, hazard ratio; NBNC, non‐HBV and non‐HCV.

^a^ Available imaging tests for response assessment in 82 patients.

#### 3.4.2 On‐treatment AFP

**FIGURE 3:**
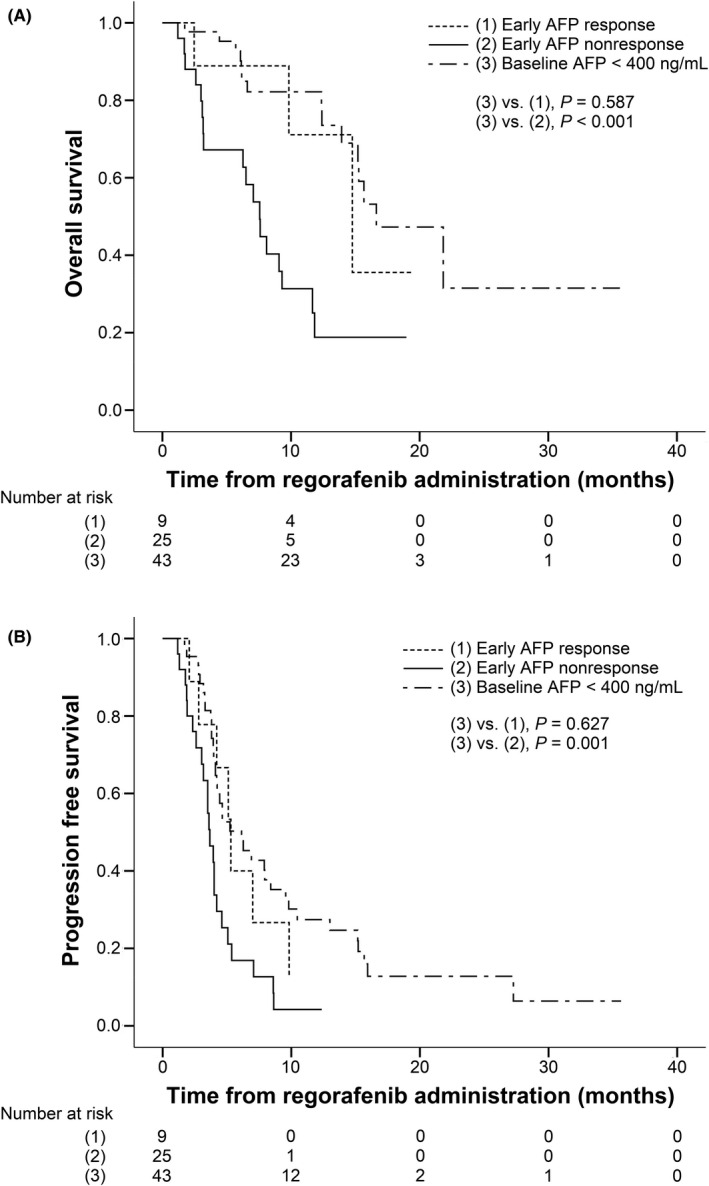
Survival outcomes of regorafenib according to baseline AFP level and the extent of AFP reduction after 4 and 8 weeks (*n* = 77). Early AFP response was defined as baseline AFP ≥ 400 ng/ml combined with >10% reduction from baseline at treatment week 4 or >20% from baseline at treatment week 8. Early AFP nonresponse was defined as baseline AFP ≥ 400 ng/ml but with increment or insufficient reduction to qualify for early AFP response. (A) Overall survival with regorafenib and (B) progression‐free survival with regorafenib

Since pretreatment AFP was the major determinant of treatment outcomes, we further evaluated the association between dynamic AFP change and treatment responses. Seventy‐seven patients with AFP data available after 4–8 weeks of regorafenib administration were studied to elucidate whether an early decline in AFP levels was associated with better treatment outcomes. Of the 77 patients, 43 patients possessing a baseline AFP < 400 ng/ml, 9 patients had an early AFP response (defined as baseline AFP ≥ 400 ng/ml and having AFP > 10% reduction after 4 weeks or >20% reduction after 8 weeks of treatment), and 25 patients had early AFP nonresponse (defined as baseline AFP ≥ 400 ng/ml but with increment or insufficient reduction to qualify for early AFP response). Patients with early AFP nonresponse exhibited a markedly shorter median OS, median PFS, and lower DCR than those who had baseline AFP < 400 ng/ml (7.6 vs. 16.6 months, *p* < 0.001; 3.7 vs. 6.1 months, *p* = 0.001; 20.0% vs. 62.8%, *p* = 0.001, respectively). In contrast, patients who reached early AFP response demonstrated a comparable median OS, median PFS, and DCR to subjects with baseline AFP < 400 ng/ml (14.8 vs. 16.6 months, *p* = 0.587; 5.3 vs. 6.1 months, *p* = 0.627; 55.6% vs. 62.8%, *p* = 0.719, respectively; Figure 3A,B), even though only three patients (33%) had follow‐up AFP levels lower than 400 ng/ml. By multivariate analysis, early AFP nonresponse, compared with baseline AFP < 400 ng/ml, was significantly associated with worse OS (HR, 3.35; *p* = 0.002), worse PFS (HR, 2.13; *p* = 0.024), and a lower DCR (OR, 0.04; *p* < 0.001). In contrast, patients with early AFP response showed insignificant differences in OS (HR, 1.32; *p* = 0.662), PFS (HR, 0.95; *p* = 0.905), and the DCR (OR, 0.76; *p* = 0.731) compared to that of those with baseline AFP < 400 ng/ml, despite possessing high baseline AFP levels (Table S6).

#### 3.4.3 Prognostic implications of hand‐foot skin reaction

The patients who experienced hand‐foot skin reaction (HFSR) tended to have better OS (median 14.8 months [95% CI, 10.9–18.6] vs. 7.1 months [95% CI, 3.4–10.7]; *p* = 0.059; Figure S3). In the multivariate analysis, HFSR was predictive of better OS (HR = 0.37, 95% CI, 0.19–0.74; *p* = 0.005; Table S7).

### 3.5 Safety

Sixty‐nine patients (80.2%) who received regorafenib had at least one adverse event. The most common adverse events were HFSR (44.2%), diarrhea (36.0%), fatigue (29.1%), and elevated aminotransferase (17.4%). The most frequently reported grade 3 or higher adverse events were elevated blood bilirubin (8.1%), HFSR (3.5%), and elevated aminotransferase (3.5%). Adverse events led to regorafenib discontinuation in eight patients (9.3%) owing to liver function worsening and fatigue. In addition, we recorded adverse events during sorafenib treatment. HFSR was also the most common adverse event (77.9%). Most adverse events occurred more frequently on sorafenib treatment than on regorafenib therapy; however, fatigue, blood bilirubin elevation, and proteinuria were more common during regorafenib treatment (29.1%, 17.4%, and 17.4%, respectively) than during sorafenib therapy (17.4%, 10.5%, and 4.7%, respectively). The majority of adverse events could be managed by dose modifications or transient discontinuation of treatment (Table 4).

**TABLE 4:** Adverse events during sorafenib and regorafenib therapy

	All		Sorafenib		Regorafenib	
	Any grade, *n* (%)	Grade ≥ 3, *n* (%)	Any grade, *n* (%)	Grade ≥ 3, *n* (%)	Any grade, *n* (%)	Grade ≥ 3, *n* (%)
Any adverse event	82 (95.3)	24 (27.9)	81 (94.2)	13 (15.1)	69 (80.2)	14 (16.3)
Hand‐foot skin reaction	69 (80.2)	13 (15.1)	67 (77.9)	11 (12.8)	38 (44.2)	3 (3.5)
Diarrhea	52 (60.5)	4 (4.7)	42 (48.8)	2 (2.3)	31 (36.0)	1 (1.2)
Fatigue	33 (38.4)	1 (1.2)	15 (17.4)	0	25 (29.1)	1 (1.2)
Aminotransferase elevation	29 (33.7)	4 (4.7)	20 (23.3)	1 (1.2)	15 (17.4)	3 (3.5)
Nausea/Vomiting	20 (23.3)	0 (0)	15 (17.4)	0 (0)	9 (10.5)	0 (0)
Alopecia	18 (20.9)	0 (0)	14 (16.3)	0 (0)	7 (8.1)	0 (0)
Blood bilirubin elevation	17 (19.8)	7 (8.1)	9 (10.5)	2 (2.3)	15 (17.4)	7 (8.1)
Proteinuria	15 (17.4)	0 (0)	4 (4.7)	0 (0)	15 (17.4)	0 (0)
Hypertension	10 (11.6)	0 (0)	8 (9.3)	0 (0)	2 (2.3)	0 (0)
Skin rash	5 (5.8)	0 (0)	5 (5.8)	0 (0)	2 (2.3)	0 (0)
Mucositis	4 (4.7)	0 (0)	4 (4.7)	0 (0)	0 (0)	0 (0)
Insomnia	3 (3.5)	0 (0)	3 (3.5)	0 (0)	0 (0)	0 (0)
Anorexia	1 (1.2)	0 (0)	0 (0)	0 (0)	1 (1.2)	0 (0)
Adverse events leading to drug discontinuation	8 (9.3)	—	0 (0)	—	8 (9.3)	—
Liver function worsening	7 (8.1)	—	0 (0)	—	7 (8.1)	—
Fatigue	1 (1.2)	—	0 (0)	—	1 (1.2)	—

## 4 DISCUSSION

Regorafenib has been approved as a second‐line systemic therapy for advanced HCC after the failure of sorafenib in Taiwan since June 2019. In the current study, we evaluated the efficacy and safety of regorafenib for patients whose HCC progressed during sorafenib treatment. We disclosed that the median OS, median PFS, and median TTP of regorafenib were 12.4, 4.2, and 4.2 months, respectively, which were comparable to the results of the phase III RESORCE trial and prior real‐world reports. In the trial, the median OS, median PFS, and median TTP by RECIST v1.1 were 10.6, 3.4, and 3.9 months, respectively.^8^ In a meta‐analysis including the RESORCE trial and seven nonrandomized studies with a total of 809 patients using regorafenib after sorafenib failure, the median OS and PFS were 11.1 and 3.2 months, respectively.^15^ Two subsequent retrospective studies from Korea also observed similar efficacy outcomes.^16^, ^17^ The median OS after sorafenib discontinuation in the current study was 15.2 months, which was remarkably longer than that in patients who received sorafenib alone (3.8 months, *p* < 0.001).^14^ Similar results were observed even after matching age, sex, Child–Pugh status, BCLC stage, and AFP level at the time of sorafenib discontinuation (15.3 vs. 5.2 months, *p* < 0.001), which indicated the survival benefit of regorafenib on uHCC after progression with sorafenib.

Nevertheless, a substantially lower ORR (4.7%) and DCR (44.2%) were observed in the current study than in the RESORCE trial (ORR: 7.7%, DCR: 66%). This difference might in part be due to the irregular interval of tumor assessment (6–12 weeks) in the real‐world setting compared to that in the strict clinical trial setting (RESORCE trial: every 6 weeks for the first 32 weeks). In fact, most of the patients enrolled in our study underwent response assessment with an interval of 10–12 weeks, which potentially led to underestimation of the ORR and DCR. Thus, the interpretation of tumor response in a retrospective study should be treated with caution. Notably, despite the relatively inferior response rate compared with that observed in the clinical trial, equally good survival rates were indicated in our patient cohort. The combination of anticancer modalities during tyrosine kinase inhibitor (TKI) treatment or rescue therapy after regorafenib failure highlighted the importance of multidisciplinary treatment strategies in clinical settings.

In our study, HCV infection, ALBI grade, macrovascular invasion, HFSR, and baseline AFP were independent prognostic factors for OS with regorafenib. Lower ALBI grades and HFSR have been identified as predictive factors for better overall survival before or during regorafenib therapy in previous reports.^18^, ^19^ The influence of underlying etiology on long‐term outcomes with systemic therapy remains controversial. One recent meta‐analysis observed no impact of etiology (viral etiologies vs. nonviral etiologies) in OS among uHCC patients treated with tyrosine kinase inhibitor/anti‐vascular endothelial growth factor.^20^ However, the trials included for analyses did not contain the RESORCE trial, which might lead to a poor applicability of the conclusion to regorafenib therapy. Possessing similar targets of tyrosine kinases with regorafenib, sorafenib exhibited greater OS benefit compared with placebo in patients with HCV (HCV positive vs. negative; HR [sorafenib/placebo], 0.47 vs. 0.81) in the SHARP trial.^21^ Notably, although HCV infection remained a prognostic factor for better OS in the sorafenib group based on the univariate analysis (HR, 0.707; *p* = 0.026), it was not a strong predictor in the multivariate analysis (HR, 0.710; *p* = 0.051). Conversely, in the RESORCE trial, greater regorafenib benefit in OS, PFS, and TTP was observed in patients without HCV infection. The reason for the contradictory results remains elusive, but it may be attributed to unknown confounders between groups with different etiologies. In the current study, HCV infection is an independent factor predictive of better OS and DCR compared with nonviral etiologies in the multivariate analysis. One possible mechanism is the upregulation of RAF‐1 induced by HCV, that is inhibited by regorafenib.^22^ Nonetheless, further investigation to elucidate this phenomenon is warranted.

The value of baseline AFP was also reported to be a predictor for survival with regorafenib; Yoo et al. from Korea observed that an AFP level ≥400 ng/ml was significantly related to OS (HR, 1.48) and PFS (HR, 1.30),^17^ while Lee et al. only found an independent association between AFP > 400 ng/ml and OS (HR, 5.9).^16^ In the current study, baseline serum AFP ≥ 400 ng/ml was independently associated with not only worse OS but also worse PFS and a lower DCR with regorafenib. Although patients possessing baseline AFP ≥ 400 ng/ml exhibited a markedly shorter median OS and PFS (8.1 and 3.7 months, respectively) compared with their counterparts (15.7 and 4.6 months, respectively), the survival outcome appeared to be comparable with those observed in the phase III REACH‐2 trial (median OS, 8.5 months; median PFS, 2.8 months), which incorporated 197 HCC patients who had AFP levels of 400 ng/ml or higher and received ramucirumab after sorafenib failure.^11^

Interestingly, because an early AFP response has been observed to predict a better treatment outcome in patients who received sorafenib and immune checkpoint inhibitors for advanced HCC,^23^, ^24^, ^25^, ^26^ we evaluated whether early AFP decline could serve as a favorable surrogate marker for regorafenib, especially for patients with higher baseline AFP levels. We observed that patients with poor early AFP response had a worse OS, PFS, and DCR compared to those with baseline AFP < 400 ng/ml. Nevertheless, patients with early AFP response exhibited a comparable OS, PFS, and DCR in comparison with those who had baseline AFP < 400 ng/ml. We demonstrated that achieving an early AFP response (>10% reduction after 4 weeks or >20% reduction after 8 weeks of treatment) could early identify the responders to regorafenib among the traditional poor responders (baseline AFP ≥ 400 ng/ml). The algorithm might help us to avoid unnecessary treatment and adverse events and seek other rescue therapies as early as possible, such as immune checkpoint inhibitors.^12^, ^13^

The safety profiles of regorafenib in this study were similar to those reported in the RESORCE trial and in previous real‐world studies, and no unpredictable toxicity was observed.^16^, ^17^, ^27^ However, in our study, the incidences of adverse events were substantially lower than those observed in the RESORCE trial (e.g., HFSR, 44% vs. 53%; diarrhea, 36% vs. 41%; fatigue, 29% vs. 40%, respectively). The frequency of grade 3 or higher adverse events was also lower in the present study. This might be ascribed to the lower starting dose allowed in our clinical practice and precautions taken to prevent adverse events that have developed during sorafenib therapy. The strategy preserved tolerability without compromising treatment efficacy. The current findings validate the safety and tolerability of regorafenib for uHCC patients after sorafenib failure in the clinical setting.

There were several limitations in our study. This study was conducted on a retrospective basis, which may have led to unintentional biases in patient selection and the assessment of safety and treatment outcomes. Unlike clinical trials, heterogeneous therapeutic strategies in clinical settings, such as concurrent therapies with sorafenib and regorafenib and varied salvage treatments following regorafenib discontinuation, might confound the interpretation of efficacy, safety, and potential prognostic factors of regorafenib. We failed to obtain the viremic status of anti‐HCV seropositive patients, who showed a better survival benefit than those with nonviral hepatitis etiology. In the era of directly acting antivirals, the current study reinforced the importance of active HCV eradication in HCC patients in terms of long‐term survival.^14^, ^28^, ^29^ Additionally, the patient number of early AFP response group was relatively small and powerless statistically. Further real‐world studies to investigate the impact of AFP response with regorafenib are needed.

## 5 CONCLUSIONS

This real‐world study verified the tolerability and efficacy of regorafenib in Taiwanese patients who had uHCC and experienced tumor progression with first‐line sorafenib therapy. Patients with baseline AFP < 400 ng/ml had better treatment outcomes than their counterparts. Despite possessing high baseline AFP levels, patients with early AFP response might benefit more from regorafenib rescue therapy.

## CONFLICT OF INTEREST

Ming‐Lung Yu has served as a speaker for AbbVie, Abbott, Bristol Myers Squibb, Gilead, Merck, a consultant for AbbVie, Abbott, Bristol Myers Squibb, Gilead, Merck, and PharmaEssentia, and has received research funding from AbbVie, Abbott, Bristol Myers Squibb, Gilead, and Merck. Chung‐Feng Huang has served as a speaker for AbbVie, Bristol Myers Squibb, Gilead, Merck, and Roche. Jee‐Fu Huang has served as a speaker for AbbVie, Bristol Myers Squibb, Gilead, Merck, Sysmex, Roche, a consultant for Roche, Bristol Myers Squibb, Gilead, Merck, Sysmex, PharmaEssentia, and Polaris Pharmaceuticals. This study was supported partially by grants from Kaohsiung Medical University (KMU‐KI110002, MOST 108‐2314‐B‐037 ‐066 ‐MY3, MOST 109‐2314‐B‐037‐044), Kaohsiung Medical University Hospital (KMUH109‐9R06, KMUH‐DK(C)110011, KMUH‐DK(C)110004, KMUH109‐9R05), Center for Cancer Research (KMU‐TC108A04‐3, KMU‐TC109A04), Center for Cancer Research KMU Global Networking Talent Plan Grant (105KMUOR08), and Center for Liquid Biopsy and Cohort Research (KMU‐TC108B06, KMU‐TC109B05, KMU‐TC108B07, KMU‐DK109002).

## ETHICAL APPROVAL STATEMENT

All procedures performed in studies involving human participants were in accordance with the ethical standards of Institutional Review Board of Kaohsiung Medical University Chung‐Ho Memorial Hospital (IRB Number: KMUHIRB‐E(I)‐20200127) and with the 1964 Helsinki declaration and its later amendments or comparable ethical standards.

## Supporting information

Fig S1

Fig S2A

Fig S2B

Fig S3

## Data Availability

The data that support the findings of this study are not publicly available due to their containing information that could compromise the privacy of research participants but are available from the corresponding authors [Yu ML and Huang CF] upon reasonable request.
