# Supplementary figures and images for: Regorafenib for Taiwanese patients with unresectable hepatocellular carcinoma after sorafenib failure: Impact of alpha‐fetoprotein levels
Source: Cancer Med. 2021 Nov 16;11(1):104–16. doi: 10.1002/cam4.4430 (PMC8704159; doi:10.1002/cam4.4430)

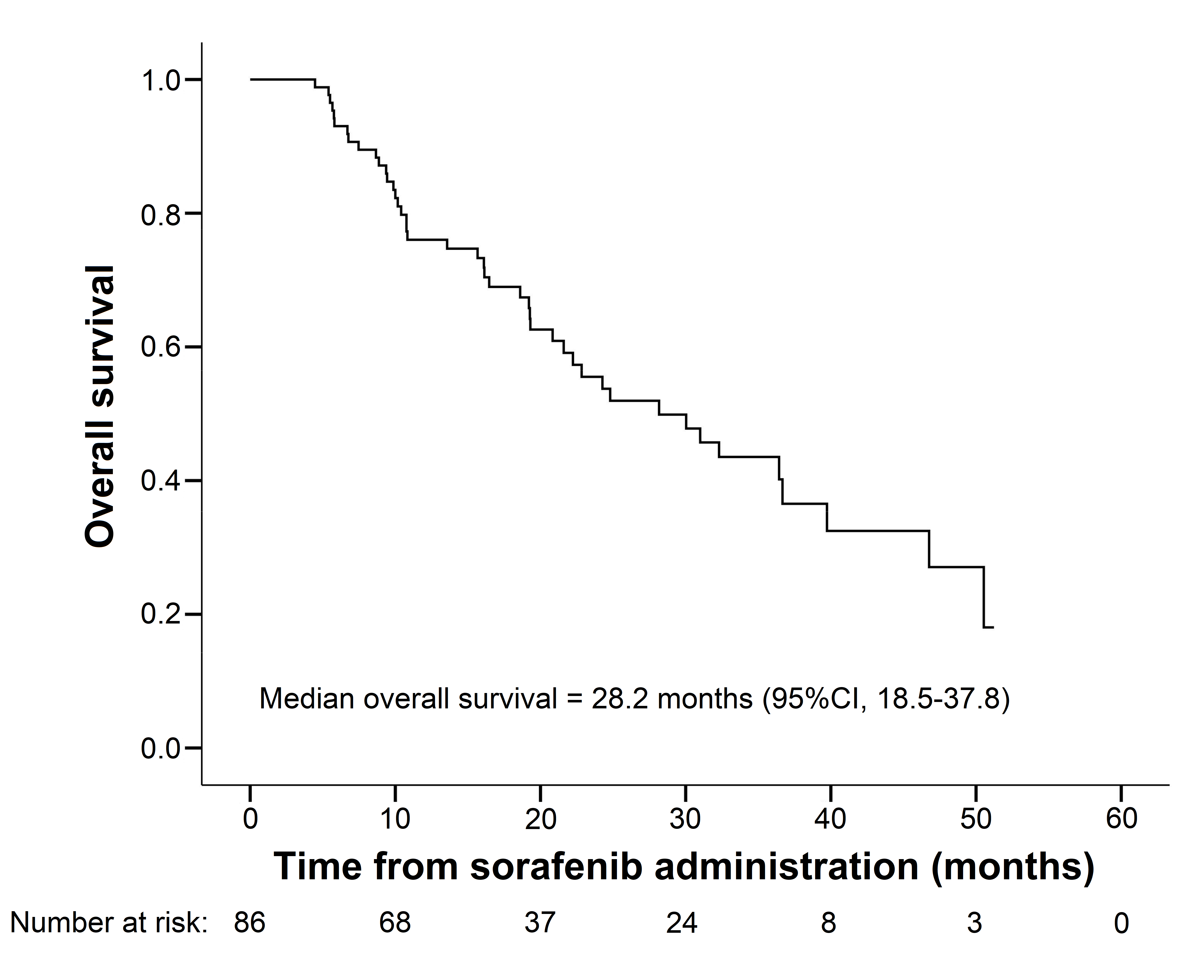

Supplement: Supplementary file 1 — Fig S1 [file CAM4-11-104-s002.tif]

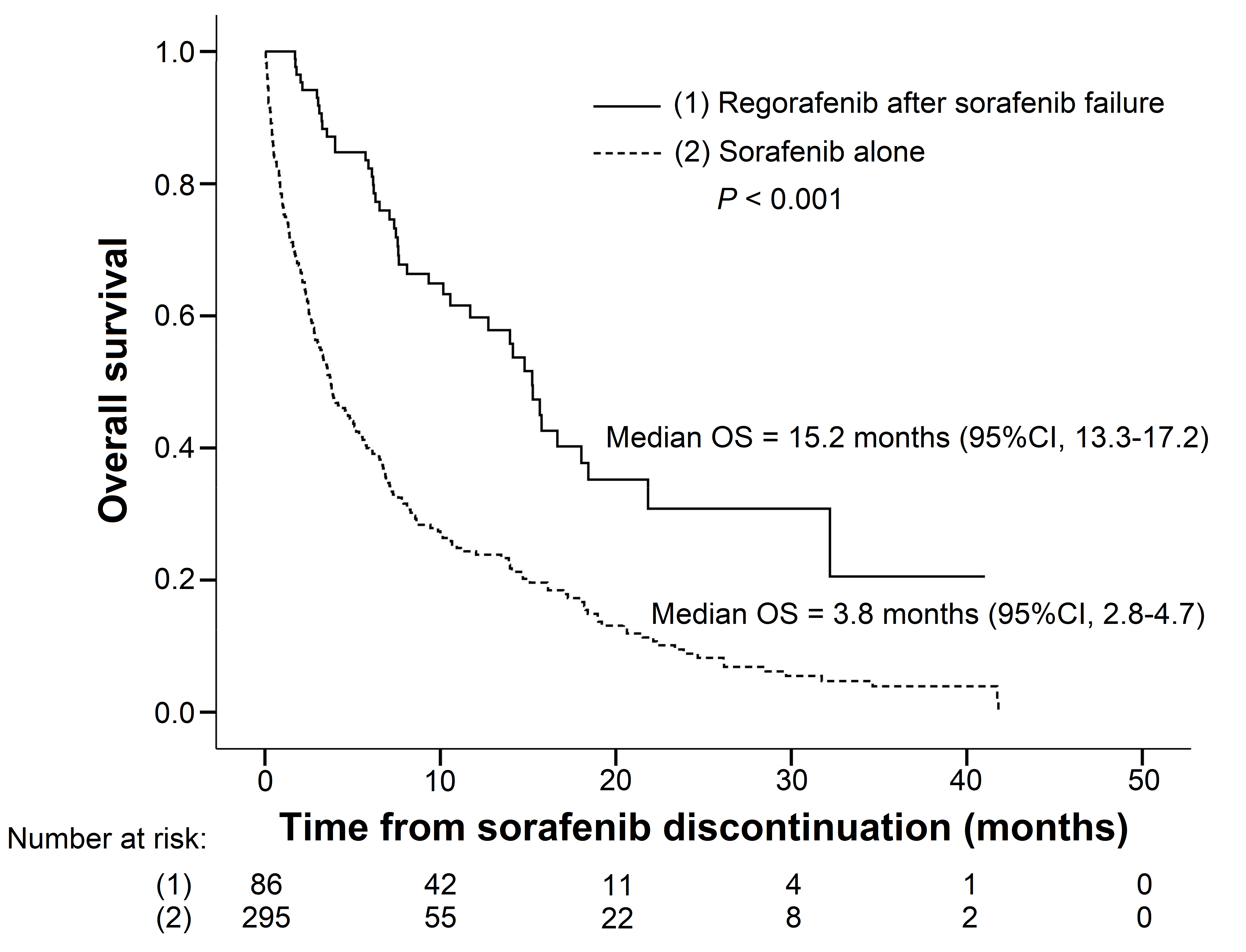

Supplement: Supplementary file 2 — Fig S2A [file CAM4-11-104-s001.tif]

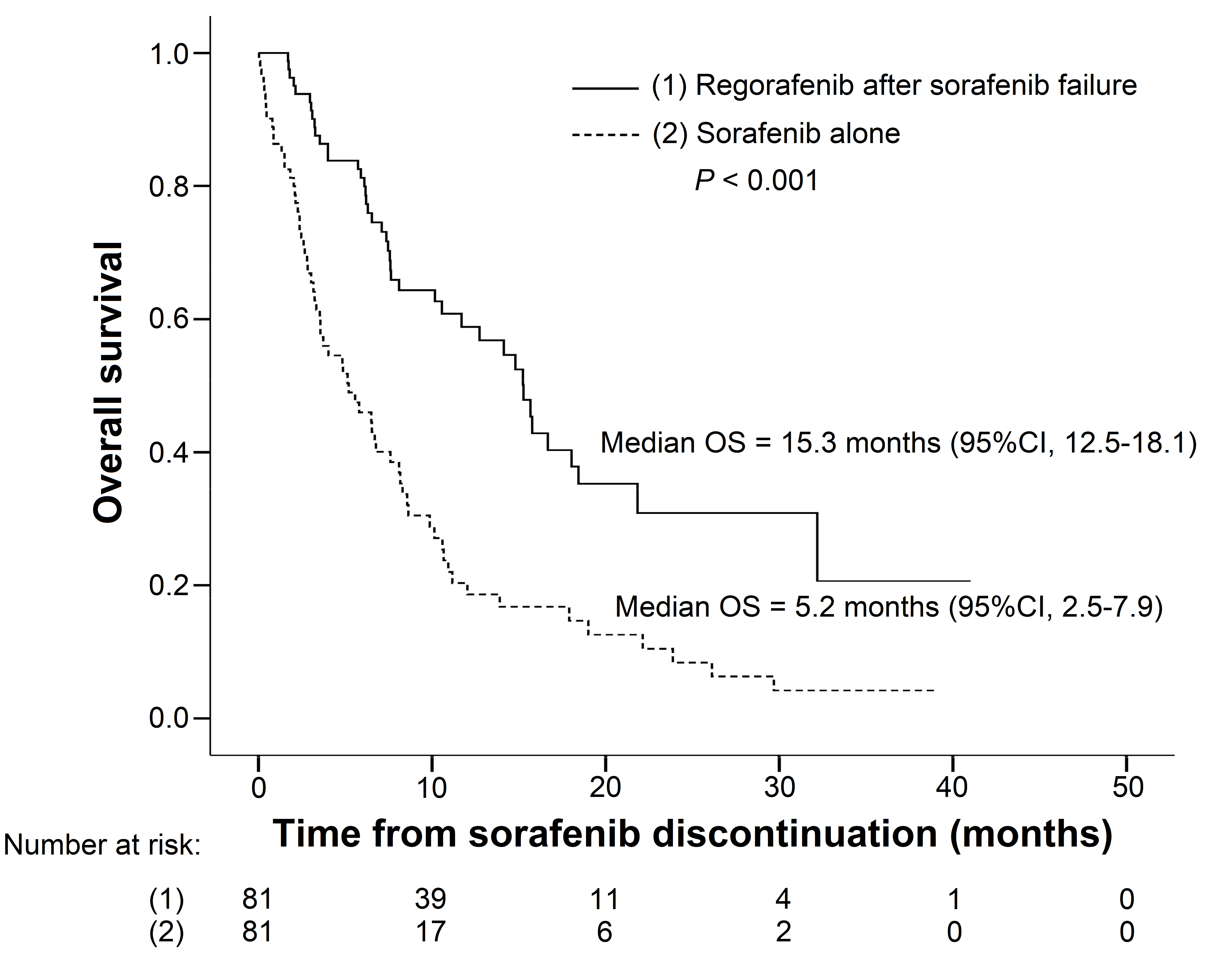

Supplement: Supplementary file 3 — Fig S2B [file CAM4-11-104-s003.tif]

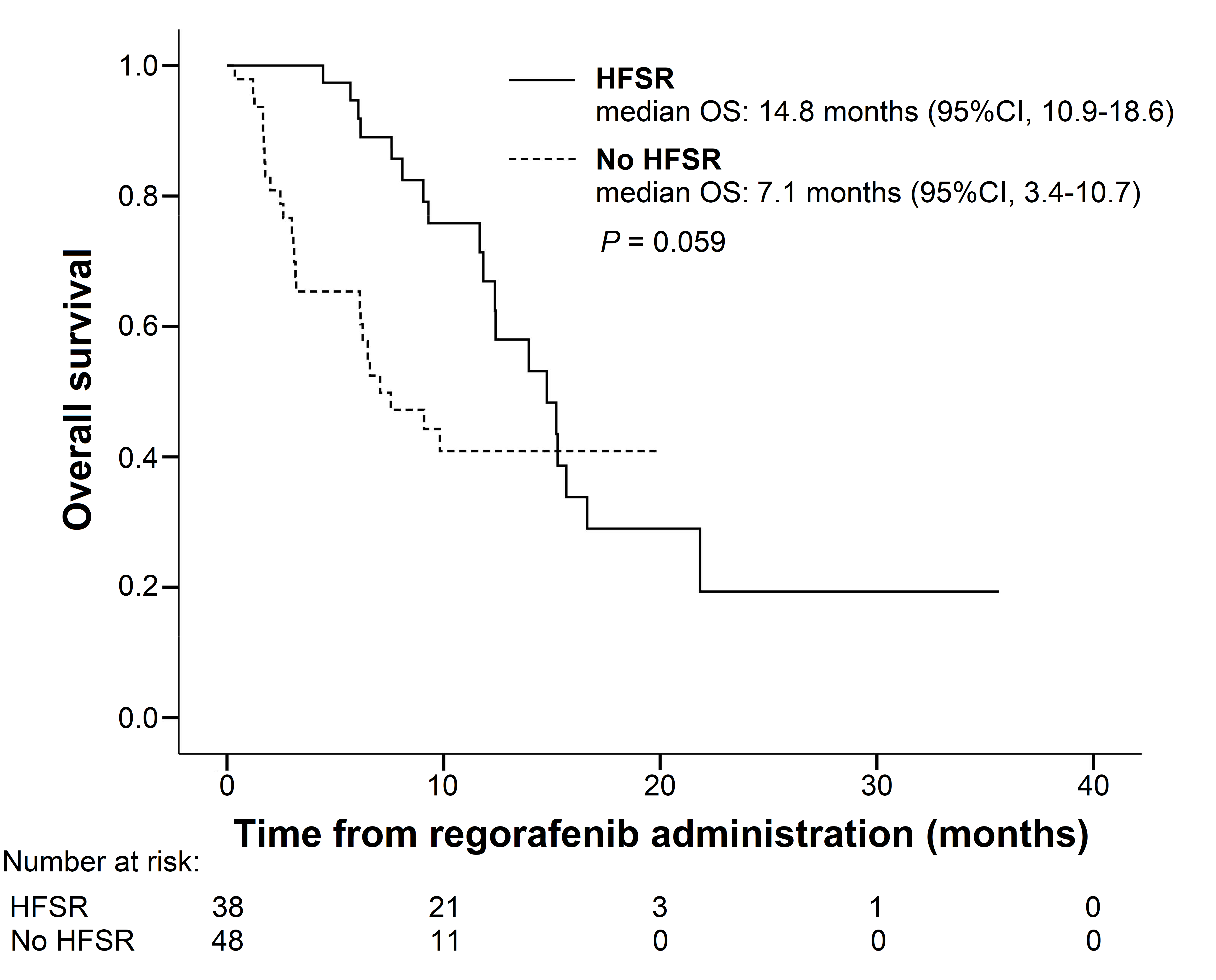

Supplement: Supplementary file 4 — Fig S3 [file CAM4-11-104-s004.tif]
