# Supplementary material for: Regorafenib for Taiwanese patients with unresectable hepatocellular carcinoma after sorafenib failure: Impact of alpha‐fetoprotein levels
Source: Cancer Med. 2021 Nov 16;11(1):104–16. doi: 10.1002/cam4.4430 (PMC8704159; doi:10.1002/cam4.4430)
Supplement: Supplementary file 5 [file CAM4-11-104-s005.docx]

**Supplementary Figure 1. Overall survival from the initiation of sorafenib administration.**

**Supplementary Figure 2.** **Overall survival (OS) after sorafenib discontinuation in (1) patients treated with regorafenib after sorafenib failure and (2) patients treated with sorafenib alone.** (A) Before propensity score matching, (B) after propensity score matching.

**Supplementary Figure 3. Overall survival with regorafenib based on the occurrence of hand-foot skin reaction (HFSR).**

Supplementary Table 1. Characteristics at the time of sorafenib discontinuation in patients receiving regorafenib after sorafenib failure (Sorafenib-regorafenib group) and patients receiving sorafenib alone (Sorafenib only group).

| Characteristics | Sorafenib-regorafenib group N= 81 | Sorafenib only group N=81 |
| --- | --- | --- |
| Age (years), median (range) | 65.0 (38-88) | 65.0 (26-83) |
| Male, n (%) | 64 (79.0) | 60 (74.1) |
| Etiology, n (%) |  |  |
| Hepatitis B | 39 (48.1) | 32 (39.5) |
| Hepatitis C | 23 (28.4) | 26 (32.1) |
| Both hepatitis B and C | 6 (7.4) | 4 (4.9) |
| Non-hepatitis B and C | 13 (16.1) | 19 (23.5) |
| Child-Pugh class, n (%) |  |  |
| A | 81 (100) | 77 (95.1) |
| BCLC stage, n (%) |  |  |
| B | 7 (8.6) | 8 (9.9) |
| C | 74 (91.4) | 73 (90.1) |
| AFP ≥ 400 ng/mL, n (%) | 38 (46.9) | 46 (56.8) |
| Sorafenib discontinuation due to disease progression, n (%) | 81 (100) | 67 (82.7) |
| Subsequent treatment, n (%) | 34 (42.0) | 20 (24.7) |

Note: BCLC stage, Barcelona clinic liver cancer stage; AFP, alpha-fetoprotein.

Supplementary Table 2. Factors associated with overall survival (OS) on regorafenib

|  | Univariate | | Multivariate | |
| --- | --- | --- | --- | --- |
|  | HR (95% CI) | *P* value | HR (95% CI) | *P* value |
| Age ≥ 65 years | 1.34 (0.72-2.47) | 0.357 |  |  |
| Male | 1.01 (0.51-2.01) | 0.975 |  |  |
| Etiology |  |  |  |  |
| NBNC |  | Reference |  | Reference |
| HBV | 0.81 (0.37-1.77) | 0.592 | 0.46 (0.19-1.10) | 0.081 |
| HCV | 0.55 (0.22-1.34) | 0.187 | 0.35 (0.13-0.94) | 0.037 |
| HBV + HCV | 0.15 (0.02-1.19) | 0.072 | 0.16 (0.02-1.33) | 0.090 |
| ALBI score |  |  |  |  |
| Grade 1 |  | Reference |  | Reference |
| Grade 2 | 3.12 (1.70-5.73) | < 0.001 | 3.75 (1.95-7.20) | < 0.001 |
| BCLC stage |  |  |  |  |
| Stage B |  | Reference |  |  |
| Stage C | 1.43 (0.56-3.65) | 0.451 |  |  |
| Largest tumor size ≥ 5cm | 1.61 (0.88-2.95) | 0.123 |  |  |
| Macrovascular invasion | 2.05 (1.12-3.72) | 0.019 | 2.03 (1.06-3.86) | 0.032 |
| Extrahepatic metastasis | 1.47 (0.81-2.69) | 0.208 |  |  |
| AFP ≥ 400 ng/mL | 2.95 (1.57-5.52) | 0.001 | 2.82 (1.36-5.84) | 0.005 |
| Concurrent treatment with regorafenib | 0.71 (0.34-1.49) | 0.364 |  |  |
| TTP on sorafenib ≥ median | 0.52 (0.28-0.95) | 0.034 | 0.64 (0.33-1.26) | 0.196 |

Note: NBNC, non-HBV and non-HCV; HBV, hepatitis B virus; HCV, hepatitis C virus; ALBI score, albumin-bilirubin score; BCLC stage, Barcelona clinic liver cancer stage; AFP, alpha-fetoprotein; TTP, time to progression; HR, hazard ratio; 95% CI, 95% confidence interval.

Supplementary Table 3. Factors associated with of progression-free survival (PFS) on regorafenib

|  | Univariate | | Multivariate | |
| --- | --- | --- | --- | --- |
| Predictor | HR (95% CI) | *P* value | HR (95% CI) | *P* value |
| Age ≥ 65 years | 1.40 (0.87-2.27) | 0.165 |  |  |
| Male | 1.71 (0.97-3.03) | 0.066 | 1.47 (0.79-2.75) | 0.224 |
| Etiology |  |  |  |  |
| NBNC |  | Reference |  | Reference |
| HBV | 1.01 (0.55-1.88) | 0.969 | 1.08 (0.55-2.13) | 0.814 |
| HCV | 0.51 (0.25-1.03) | 0.061 | 0.58 (0.27-1.27) | 0.174 |
| HBV + HCV | 0.31 (0.10-0.94) | 0.039 | 0.34 (0.10-1.14) | 0.081 |
| ALBI score |  |  |  |  |
| Grade 1 |  | Reference |  |  |
| Grade 2 | 1.34 (0.84-2.13) | 0.223 |  |  |
| BCLC stage |  |  |  |  |
| Stage B |  | Reference |  |  |
| Stage C | 1.50 (0.74-3.03) | 0.257 |  |  |
| Largest tumor size ≥ 5cm | 2.01 (1.23-3.26) | 0.005 | 1.36 (0.77-2.41) | 0.287 |
| Macrovascular invasion | 1.59 (1.00-2.52) | 0.050 | 1.26 (0.75-2.12) | 0.376 |
| Extrahepatic metastasis | 1.84 (1.15-2.94) | 0.011 | 1.99 (1.21-3.28) | 0.007 |
| AFP ≥ 400 ng/mL | 2.03 (1.25-3.28) | 0.004 | 1.82 (1.04-3.16) | 0.035 |
| Concurrent treatment with regorafenib | 0.92 (0.55-1.56) | 0.764 |  |  |
| TTP on sorafenib ≥ median | 0.64 (0.40-1.02) | 0.058 | 1.06 (0.62-1.81) | 0.841 |

Note: NBNC, non-HBV and non-HCV; HBV, hepatitis B virus; HCV, hepatitis C virus; ALBI score, albumin-bilirubin score; BCLC stage, Barcelona clinic liver cancer stage; AFP, alpha-fetoprotein; TTP, time to progression; HR, hazard ratio; 95% CI, 95% confidence interval.

Supplementary Table 4. Factors associated with time to progression (TTP) on regorafenib

|  | Univariate | | Multivariate | |
| --- | --- | --- | --- | --- |
| Predictor | HR (95% CI) | *P* value | HR (95% CI) | *P* value |
| Age ≥ 65 years | 1.43 (0.85-2.41) | 0.174 |  |  |
| Male | 2.33 (1.18-4.60) | 0.015 | 2.01 (0.97-4.18) | 0.061 |
| Etiology |  |  |  |  |
| NBNC |  | Reference |  |  |
| HBV | 0.97 (0.51-1.85) | 0.928 | 1.15 (0.56-2.34) | 0.707 |
| HCV | 0.41 (0.19-0.88) | 0.023 | 0.51 (0.22-1.22) | 0.130 |
| HBV + HCV | 0.33 (0.11-1.04) | 0.058 | 0.39 (0.11-1.36) | 0.141 |
| ALBI score |  |  |  |  |
| Grade 1 |  | Reference |  |  |
| Grade 2 | 1.06 (0.63-1.77) | 0.834 |  |  |
| BCLC stage |  |  |  |  |
| Stage B |  | Reference |  |  |
| Stage C | 1.48 (0.70-3.13) | 0.300 |  |  |
| Largest tumor size ≥ 5cm | 1.98 (1.18-3.34) | 0.010 | 1.48 (0.83-2.63) | 0.187 |
| Macrovascular invasion | 1.48 (0.90-2.45) | 0.126 |  |  |
| Extrahepatic metastasis | 2.42 (1.45-4.03) | 0.001 | 2.76 (1.59-4.78) | < 0.001 |
| AFP ≥ 400 ng/mL | 1.86 (1.11-3.12) | 0.019 | 1.61 (0.88-2.94) | 0.120 |
| Concurrent treatment with regorafenib | 1.05 (0.60-1.82) | 0.871 |  |  |
| TTP on sorafenib ≥ median | 0.57 (0.35-0.94) | 0.027 | 0.91 (0.51-1.63) | 0.755 |

Note: NBNC, non-HBV and non-HCV; HBV, hepatitis B virus; HCV, hepatitis C virus; ALBI score, albumin-bilirubin score; BCLC stage, Barcelona clinic liver cancer stage; AFP, alpha-fetoprotein; TTP, time to progression; HR, hazard ratio; 95% CI, 95% confidence interval.

Supplementary Table 5. Factors associated with disease control rate on regorafenib, n=82

|  | Univariate | | Multivariate | |
| --- | --- | --- | --- | --- |
|  | OR (95% CI) | *P* value | OR (95% CI) | *P* value |
| Age ≥ 65 years | 0.88 (0.36-2.12) | 0.767 |  |  |
| Male | 0.58 (0.20-1.68) | 0.317 |  |  |
| Etiology |  |  |  |  |
| NBNC |  | Reference |  | Reference |
| HBV | 1.67 (0.45-6.29) | 0.445 | 3.12 (0.64-15.29) | 0.161 |
| HCV | 3.85 (0.95-15.66) | 0.060 | 5.72 (1.12-29.11) | 0.036 |
| HBV + HCV | 13.75 (1.21-156.65) | 0.035 | 16.53 (0.94-289.88) | 0.055 |
| ALBI score |  |  |  |  |
| Grade 1 |  | Reference |  |  |
| Grade 2 | 0.53 (0.21-1.32) | 0.174 |  |  |
| BCLC stage |  |  |  |  |
| Stage B |  | Reference |  |  |
| Stage C | 0.26 (0.06-1.06) | 0.060 | 0.28 (0.05-1.45) | 0.129 |
| Largest tumor size ≥ 5cm | 0.47 (0.19-1.16) | 0.102 |  |  |
| Macrovascular invasion | 1.12 (0.46-2.74) | 0.799 |  |  |
| Extrahepatic metastasis | 0.28 (0.11-0.71) | 0.007 | 0.41 (0.13-1.24) | 0.114 |
| AFP ≥ 400 ng/mL | 0.25 (0.10-0.63) | 0.004 | 0.16 (0.05-0.52) | 0.002 |
| Concurrent treatment with regorafenib | 1.68 (0.63-4.49) | 0.301 |  |  |
| TTP on sorafenib ≥ median | 1.64 (0.68-3.94) | 0.268 |  |  |

Note: NBNC, non-HBV and non-HCV; HBV, hepatitis B virus; HCV, hepatitis C virus; ALBI score, albumin-bilirubin score; BCLC stage, Barcelona clinic liver cancer stage; AFP, alpha-fetoprotein; TTP, time to progression; HR, hazard ratio; 95% CI, 95% confidence interval.

Supplementary Table 6. Factors including AFP dynamics predictive of treatment outcomes after regorafenib administration †

| Variables | Univariate HR  (95% CI) | *P* value | Multivariate HR  (95%CI) | *P* value |
| --- | --- | --- | --- | --- |
| **Overall survival** |  |  |  |  |
| ALBI grade 2 (vs. grade 1) | 3.09 (1.58-6.05) | 0.001 | 3.83 (1.90-7.73) | < 0.001 |
| AFP ＜ 400 ng/mL |  | Reference |  | Reference |
| Early AFP response | 1.38 (0.40-4.84) | 0.612 | 1.32 (0.38-4.64) | 0.662 |
| Early AFP nonresponse | 4.18 (2.02-8.64) | < 0.001 | 3.35 (1.55-7.24) | 0.002 |
| TTP on sorafenib ≥ median | 0.42 (0.21-0.83) | 0.013 | 0.41 (0.19-0.87) | 0.020 |
| **Progression-free survival** |  |  |  |  |
| Extrahepatic metastasis | 1.84 (1.12-3.02) | 0.017 | 2.06 (1.21-3.50) | 0.008 |
| AFP ＜ 400 ng/mL |  | Reference |  | Reference |
| Early AFP response | 1.22 (0.53-2.79) | 0.638 | 0.95 (0.39-2.32) | 0.905 |
| Early AFP nonresponse | 2.51 (1.44-4.38) | 0.001 | 2.13 (1.11-4.10) | 0.024 |
| **Time to progression** |  |  |  |  |
| Extrahepatic metastasis | 2.22 (1.31-3.75) | 0.003 | 2.51 (1.43-4.41) | 0.001 |
| Variables | Univariate OR  (95% CI) | *P* value | Multivariate OR  (95%CI) | *P* value |
| **Disease control rate** |  |  |  |  |
| NBNC |  | Reference |  | Reference |
| HBV + HCV | 10.00 (0.86-117.02) | 0.067 | 30.73 (1.04-908.77) | 0.047 |
| BCLC stage C (vs. stage B) | 0.29 (0.07-1.21) | 0.089 | 0.11 (0.01-0.87) | 0.036 |
| AFP ＜ 400 ng/mL |  | Reference |  | Reference |
| Early AFP response | 0.74 (0.17-3.17) | 0.686 | 0.76 (0.16-3.66) | 0.731 |
| Early AFP nonresponse | 0.15 (0.05-0.47) | 0.001 | 0.04 (0.01-0.25) | < 0.001 |

Note: †n=77; AFP, alpha-fetoprotein; early AFP response defined as baseline AFP ≥ 400 ng/mL and having AFP >10% reduction at 4 weeks or >20% reduction at 8 weeks after regorafenib administration; early AFP nonresponse defined as baseline AFP ≥ 400 ng/mL but with increment or insufficient reduction to qualify for early AFP response; ALBI score, albumin-bilirubin score; HR, hazard ratio; 95% CI, 95% confidence interval.

Supplementary Table 7. Multivariate analysis for the relationship between hand-foot and overall survival with regorafenib

| Variables | Overall survival | | |
| --- | --- | --- | --- |
|  | Hazard ratio | 95% Confidence interval | *P* value |
| Etiology |  |  |  |
| NBNC |  |  | Reference |
| HBV | 0.44 | 0.18-1.05 | 0.063 |
| HCV | 0.29 | 0.11-0.79 | 0.016 |
| HBV + HCV | 0.14 | 0.02-1.21 | 0.074 |
| ALBI score |  |  |  |
| Grade 1 |  |  | Reference |
| Grade 2 | 4.66 | 2.34-9.31 | < 0.001 |
| Macrovascular invasion | 2.42 | 1.23-4.77 | 0.011 |
| AFP ≥ 400 ng/mL | 2.26 | 1.07-4.78 | 0.034 |
| TTP on sorafenib ≥ median | 0.66 | 0.33-1.32 | 0.240 |
| Hand-foot skin reaction | 0.37 | 0.19-0.74 | 0.005 |

Note: NBNC, non-HBV and non-HCV; HBV, hepatitis B virus; HCV, hepatitis C virus; ALBI score, albumin-bilirubin score; AFP, alpha-fetoprotein; TTP, time to progression.
